# Ultrasound as a Physical Elicitor to Improve Texture in Blueberry Fruit: Physiological Indicator and Transcriptomic Analysis

**DOI:** 10.3390/foods13203246

**Published:** 2024-10-12

**Authors:** Yuanyuan Hou, Yinggang Ge, Ailikemu Mulati, Yuting Yang, Jiayi Wang

**Affiliations:** National Demonstration Center for Experimental Biology Education, Xinjiang Key Laboratory of Biological Resources and Genetic Engineering, College of Life Science & Technology, Xinjiang University, Urumqi 830046, China; houyy@xju.edu.cn (Y.H.); 107552303723@stu.xju.edu.cn (Y.G.); alkam@stu.xju.edu.cn (A.M.); 13350321557@139.com (Y.Y.)

**Keywords:** blueberry fruit, fruit quality, ultrasound washing, transcriptome, lignin biosynthesis

## Abstract

Ultrasound (US) washing has been verified to improve the quality of postharvest blueberry fruit. However, its physiological and molecular mechanisms remain largely unknown. In the present study, an US with a frequency of 25 kHz and a power density of 400 W for 2 min was performed to investigate its role in impacting the quality of blueberries. The results showed that US washing improved the quality of blueberries, with a higher firmness and lignin content (*p* < 0.05) than the control. Moreover, US washing inhibited the levels of superoxide radical (O_2_^·−^) production rate and hydrogen peroxide (H_2_O_2_) content while stimulating the superoxide dismutase (SOD) and catalase (CAT) activities of the blueberry fruit. Transcriptomic analysis screened 163 differentially expressed genes (DEGs), and the key DEGs were mainly enriched in phenylpropanoid biosynthesis, flavonoid biosynthesis, and plant–pathogen interaction pathways. Furthermore, the transcription factors and the structural genes associated with lignin biosynthesis were also identified from the DEGs. More importantly, the correlation analysis revealed that firmness and lignin content were positively correlated with the expression of C4H, COMT1, and POD52 in blueberry fruit, indicating that these genes might be involved in the regulation of US-mediated lignin synthesis. The findings provide new insight into the US-enhanced quality of blueberry fruits.

## 1. Introduction

Fresh fruits and vegetables are key sources of vitamins, dietary fiber, and minerals for human consumption and are important for maintaining health. Blueberries are often consumed fresh and are popular due to their colors, flavor, and healthy nutrients, such as anthocyanins, polyphenols, and flavonoids [1]. However, fresh blueberries are susceptible to quality deterioration because of rapid softening and microbial contamination after harvest, thus leading to a reduction in freshness and short shelf life [2]. Therefore, the development of effective disinfection technology is necessary to reduce decay and improve the quality of fresh blueberry fruit.

Ready-to-eat fruits and vegetables are produced from fresh fruits and vegetables through a series of processes, such as cleaning, disinfection, cutting, and packaging. Among these processes, cleaning is a key step, which effectively removes contaminants and microorganisms, ensuring the safety of the products in the subsequent processing and stability during storage [3]. However, ordinary water washing has a very limited effect on removing microorganisms and pesticide residues and requires high water usage. Thus, seeking some more effective cleaning methods to improve the quality of fresh fruits and vegetables and reduce waste has become an urgent problem to be solved. Ultrasound (US) washing technology, as a low-cost and efficient method, has been widely applied to fruit and vegetable cleaning and preservation processes [4]. Generally, an ultrasound combined with other novel and conventional methods, such as surfactants and disinfectants, can enhance the ultrasound-based effect and be more effective and sustainable in protecting fruits and vegetables from microbial contamination [5]. The contaminants and microorganisms on the surface of horticultural products can be removed via ultrasound washing, including *Escherichia coli*, *Salmonella*, and *Listeria*. The microorganisms could be killed via cavitation of low-frequency power ultrasound through destroying cell membranes and causing intracellular material leakage [6]. For example, under the ultrasonic-assisted washing conditions with a frequency of 40 kHz, an ultrasound power density of 125.45 W/L, and a washing time of 15 min, the log reduction predictions were 5.6 and 4.7 CFU/g for *E. coli* and *L. innocua* in Chinese cabbage, respectively [7]. Additionally, in cauliflower, using 0.5% zinc acetate, 0.06% tea saponins, or 5% ethanol could effectively enhance the removal of microorganisms (total bacterial removal increased by about 2.0 log CFU/g) compared with ultrasonic cleaning alone [8,9]. Although high-frequency US treatment could affect the cells of fruits and vegetables to a certain extent, the suitable low-frequency (20–100 kHz) US has been confirmed to prolong the shelf life of several fresh fruits and vegetables [5]. For instance, in fresh-cut red cabbages, ultrasonic washing could promote phenolic accumulation, enhance sensory quality, and inhibit the growth of microorganisms by destroying their cell membranes to extend the shelf-life [10,11]. Cao et al. [12] found that ultrasonic washing (40 kHz) could reduce decay incidence and the number of microorganisms and maintain higher firmness and nutrient contents in strawberry fruit. In blueberry fruit, Zhang et al. [13] showed that ultrasound (20 kHz) washing suppressed the natural microflora growth and maintained fruit quality during storage. Additionally, 25 kHz low-frequency US washing combined with disinfectants (free chlorine [FC] at 10 ppm and peracetic acid [PAA] at 80 ppm) could significantly reduce the *Escherichia coli* O157:H7 and Salmonella Typhimurium counts and regulate antioxidant enzyme activity to eliminate reactive oxygen species (ROS), thus retaining higher quality in fresh blueberries [14]. Although the efficacy of US washing in the microorganism controlling and quality maintenance of fresh blueberries has been demonstrated, the underlying molecular mechanism of improving the quality remains unknown.

The transcriptomic analysis can used for constructing genome-wide gene expression profiles to screen key genes involved in specific physiological processes of postharvest fruits [15] and is also a powerful method to investigate the molecular regulation of postharvest fruits, such as longan fruit [16], fresh-cut apple [17], and fresh-cut lettuce [18]. It has been well-demonstrated that texture changes in fruits and vegetables are closely associated with lignin biosynthesis via phenylpropanoid metabolism. Many studies have shown that a series of key genes are involved in this process. In loquat fruit, RNA sequencing (RNA-Seq) analysis unraveled that the up-regulated genes *PAL*, *CAD*, *4CL*, *COMT*, *HCT*, *POD*, and *LAC* participated in the lignin biosynthesis pathway, thereby leading to lignification development [19,20]. Hou et al. [21] revealed that *PvPAL2/4/6*, *PvC3H2/3*, *PvC4H2/4*, *PvCAD1/2/3/4*, *PvCCR2/4*, and *PvHCT2/5/8* are key genes involved in lignin biosynthesis, which provides information for further investigating into the molecular mechanism of lignin accumulation in bamboo shoots. Thus, adopting RNA sequencing (RNA-Seq) analysis to identify the key genes of lignin synthesis in postharvest fruits is of great significance for exploring the potential regulatory mechanisms of fruit texture changes. However, no such comprehensive studies are available to elucidate the molecular regulatory mechanisms underlying texture change induced by US washing in blueberry fruit.

Therefore, we supposed that US washing extending the shelf life of blueberry fruit might be due to its role in texture regulation, which is closely related to lignin change. In this work, an RNA-Seq analysis of blueberry fruit was conducted to identify the potential candidate genes and pathways associated with texture changes, along with the determination of ROS metabolism, contributing to obtaining new insight into the physiological and molecular events of quality in blueberry fruit induced by US washing. Our results establish a basis for further study on the regulatory mechanism of US washing to improve the quality of postharvest fruits.

## 2. Materials and Methods

### 2.1. Materials and Treatments

Blueberry (Joyvio, Beijing, China) fruit was obtained from a local market with commercial maturity (firmness nearly 4.2 N), and fruits with uniform size and maturity were carefully chosen for the experiment, with no mechanical injury or disease. Before treatment, the fruits were thoroughly rinsed in tap water for 30 s to eliminate dirt or impurities and then divided into two groups randomly (every group contained 300 fruits containing three replicates). For the US washing group, the ultrasound device in this work was used according to a previous study [14], and fruits were washed under ultrasound with a frequency of 25 kHz and a power density of 400 W for 2 min at room temperature. For the control group, blueberry fruit was washed with tap water for 2 min at room temperature. After treatment, the fruit was dried naturally at room temperature for 2 h, and three replicate samples were collected and frozen in liquid nitrogen, then stored at −80 °C for further measurement. Before the blueberry fruit was frozen, firmness was measured using fresh fruit.

### 2.2. Determination of Firmness

The firmness was assayed following the method of Wang et al. [22] using fresh fruit. A penetrometer (GY-4; Aidebao, Yueqing, China) was applied to measure the fruit firmness by penetrating a depth of 10 mm, which was equipped with a cylindrical probe (3.5 mm diameter). The results were expressed as Newtons (N).

### 2.3. Determination of Lignin Content

Lignin content was detected based on the method described by Morrison [23], with some modifications. Briefly, 10 g of flesh sample was homogenized with 40 mL 95% (*v*/*v*) ethanol and centrifuged at 10,000× *g* for 10 min at 4 °C. After washing three times with 95% (*v*/*v*) ethanol, the precipitate was then washed three times with an ethanol–hexane mixture (1:2, *v*/*v*). The reaction was terminated via adding 10 mL of 2 M NaOH. Subsequently, after adding 20 mL of glacial acetic acid and 1 mL of 7.5 M hydroxylamine hydrochloride, the mixture was centrifuged. Using glacial acetic acid to dilute the 0.5 mL of supernatant to 10 mL, the absorbance at 280 nm was assayed. The results were expressed as OD280 g^−1^ on a fresh weight (FW) basis.

### 2.4. Measurement of Superoxide Radical (O_2_^·−^) Production Rate and Hydrogen Peroxide (H_2_O_2_) Content

The determinations of the O_2_^·−^ production rate and H_2_O_2_ content were performed following the description of Zuo et al. [24] using frozen samples. For the O_2_^·−^ production rate, 0.5 g of grounded powder was mixed with 2.5 mL of 50 mM sodium phosphate buffer (pH 7.8) and then centrifuged at 11,000× *g* for 10 min at 4 °C. The reaction system included 1 mL of crude extract, 1 mL of 50 mM PBS (pH 7.8), 1 mL of 10 mM hydroxylammonium chloride, 1 mL of 17 mM 4-aminobenzene sulfonic acid, and 1 mL of 7 mM α-naphthylamine. After the solution turned colorless, the absorbance at 530 nm was detected. Subsequently, 3 mL of 2 M sulfuric acid was used to dissolve the precipitate, and then the absorbance was measured at 412 nm. The results were shown as μmol min^−1^ g^−1^ FW.

For the H_2_O_2_ content assay, 0.5 g of grounded powder was mixed with 2.5 mL of pre-cooled acetone and centrifuged at 11,000× *g* for 10 min at 4 °C. The reaction system contained 0.5 mL of crude extract, 0.5 mL of 10 mM PBS (pH 7.0), and 1 mL of 1 M kalium iodidum. The absorbance was determined at 390 nm after incubation for 1 h in the dark. The H_2_O_2_ content was expressed as μmol g^−1^ FW.

### 2.5. Detection of Superoxide Dismutase (SOD) and Catalase (CAT) Activities

The activities of SOD and CAT were assayed according to a previous method [24]. For SOD analysis, 0.5 g of flesh sample was homogenized with 2.5 mL of 50 mM PBS (pH 7.8). The reaction system contained 0.1 mL crude extract, 3 mL of reaction solution containing 13 μM methionine, 63 μM nitroblue tetrazolium (NBT), 1.3 μM riboflavin, 0.1 mM EDTA, and 50 mM PBS. The absorbance was measured at 560 nm after illuminating under a light intensity of 4000 lx for 10 min. The amount of enzyme that caused a 50% inhibition of NBT reduction was defined as a unit of SOD activity. The activity of SOD was represented as U g^−1^ FW.

For CAT activity, the reaction system contained 0.1 mL crude extract and 2.9 mL of 20 mM H_2_O_2_. The absorbance was assayed at 240 nm within 3 min at 37 °C and one unit of CAT activity was defined as the amount of enzyme that caused a 0.01 change in absorbance per minute. The activity of CAT was represented as U g^−^^1^ FW.

### 2.6. RNA-Seq Analysis

Samples of blueberry fruit in both the control and US washing groups were used for RNA-Seq analysis through Personalbio Technologies Co., Ltd. (Shanghai, China). Total RNA was isolated via the RNA prep Pure Plant Plus Kit (Tiangen Biotech (Beijing) Co., Ltd., Beijing, China) based on the instructions. The NovaSeq 6000 platform (Illumina, San Diego, CA, USA) was applied to construct a sequencing library. The fastp (0.22.0) software was used for filtering the sequencing data to obtain clean data. The read count values on each gene as the original expression of the gene were compared by HTSeq (v0.9.1) statistics, and then the expression was standardized using FPKM. DESeq (v1.38.3) to analyze differentially expressed genes (DEGs) with screened conditions as follows: |log2FoldChange| > 1, and *p*-value < 0.05 [25].

### 2.7. Genes Expression Assay

The cDNA synthesis and quantitative real-time PCR (qRT-PCR) were performed by Personalbio Technologies Co. Ltd. (Shanghai, China). The relative expression of the genes was computed by the 2^−ΔΔCt^ method and the specific primers for qRT-PCR are presented in Supplementary Table S1.

### 2.8. Statistical Analysis

All experiments were performed in three biological replicates and the data were shown as the means ± standard deviation (SD). The data of different treatments were compared using the *t*-test (*p* < 0.05) in IBM SPSS Statistics 22 (SPSS Inc., Chicago, IL, USA). Principal component analysis (PCA) and correlation analysis were carried out using Origin 2024 (Microcal software), which comprehensively evaluated the influence of US on the quality of blueberry fruit.

## 3. Results

### 3.1. The Effects of US Washing on the Firmness and Lignin Content in Blueberries

**Figure 1:**
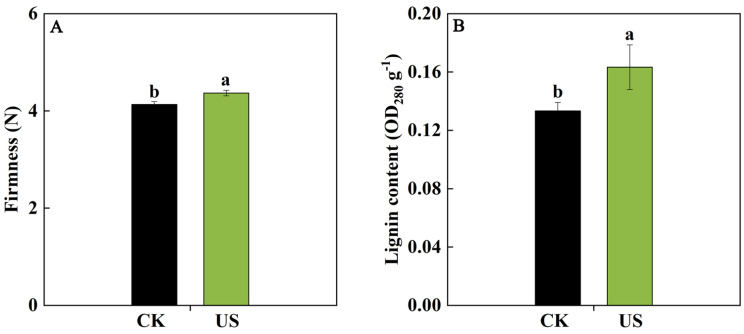
US washing enhanced firmness (**A**) and lignin content (**B**) of blueberries. CK and US represent the control group and ultrasound washing group, respectively. Vertical bars represent the SD of the mean (*n* = 3). The significant differences (*p* < 0.05) between the two groups are denoted by different letters.

As shown in Figure 1, the firmness and lignin content were determined after US treatment. US washing significantly increase the firmness and lignin content in blueberry fruit, which were 5.65% and 22.5% (*p* < 0.05) higher in the US-treated group than those in the control. These results indicated that US washing could maintain fruit firmness, which was highly associated with the higher lignin content.

### 3.2. The Effects of US Washing on the ROS Metabolism in Blueberries

**Figure 2:**
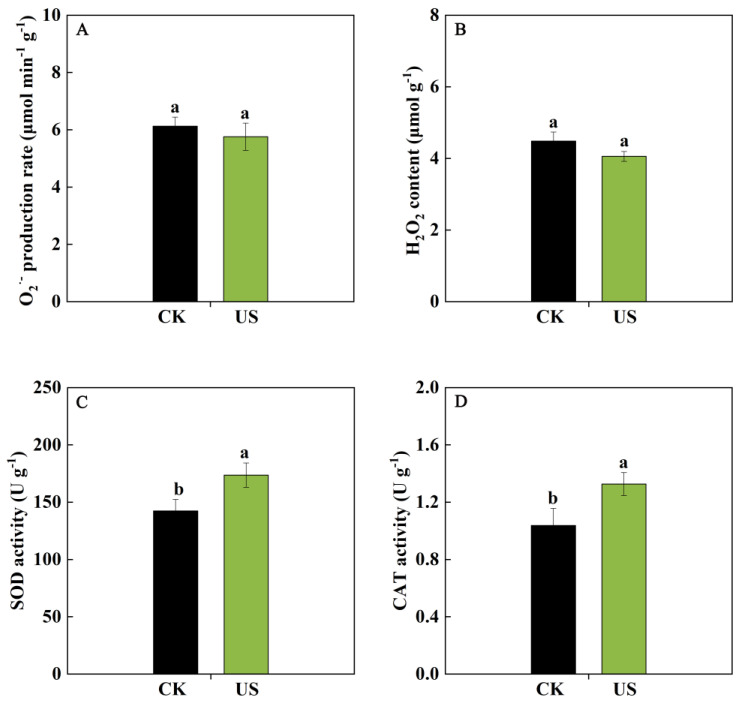
Effects of US washing on ROS metabolism of blueberry fruit. (**A**,**B**) The O_2_^·−^ production rate and H_2_O_2_ content of blueberry fruit. (**C**,**D**) The SOD and CAT activities of blueberries. CK and US represent the control group and ultrasound washing group, respectively. Vertical bars represent the SD of the mean (*n* = 3). The significant differences (*p* < 0.05) between the two groups are denoted by different letters.

Compared with the control, US washing could inhibit the levels of O_2_^·−^ and H_2_O_2_, but there were no remarkable differences (*p* > 0.05) between US washing and the control group (Figure 2A,B). In contrast, the SOD and CAT activities were markedly promoted by US washing, which were 22.01% and 27.97% (*p* < 0.05) higher than those in the control (Figure 2C,D), respectively. These results implied that US washing could reduce ROS levels via activating SOD and CAT activities in blueberries.

### 3.3. RNA-Seq Analysis of US-Improved Quality in Blueberry Fruit

**Figure 3:**
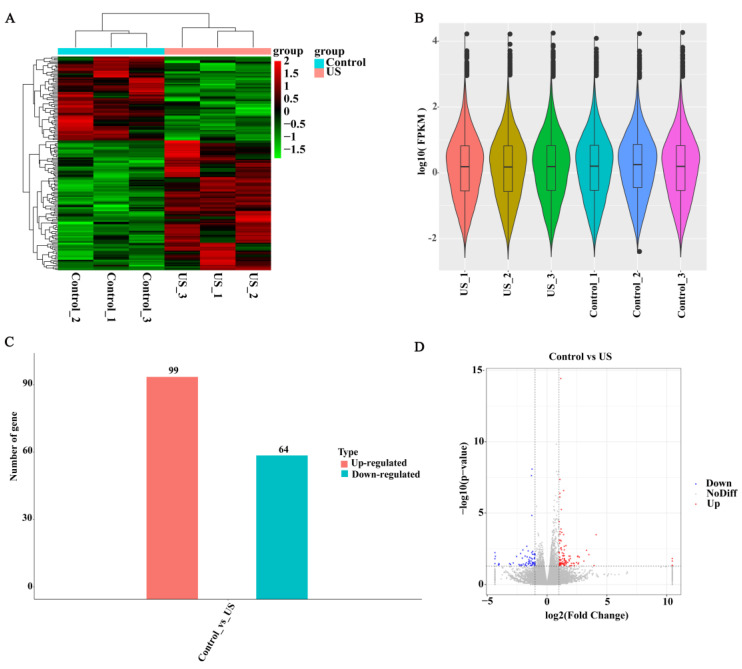
Overview of the RNA-Seq analysis of blueberry fruit. (**A**) Clustering heat map of DEGs between the two groups. (**B**) The expression range of the two groups was displayed using a violin plot. (**C**) DEGs’ number in CK vs. US. (**D**) Volcano plots revealing the DEGs in CK vs. US. US represents the ultrasound washing group. The up-regulated and down-regulated genes were represented by red and blue spots, respectively.

**Figure 4:**
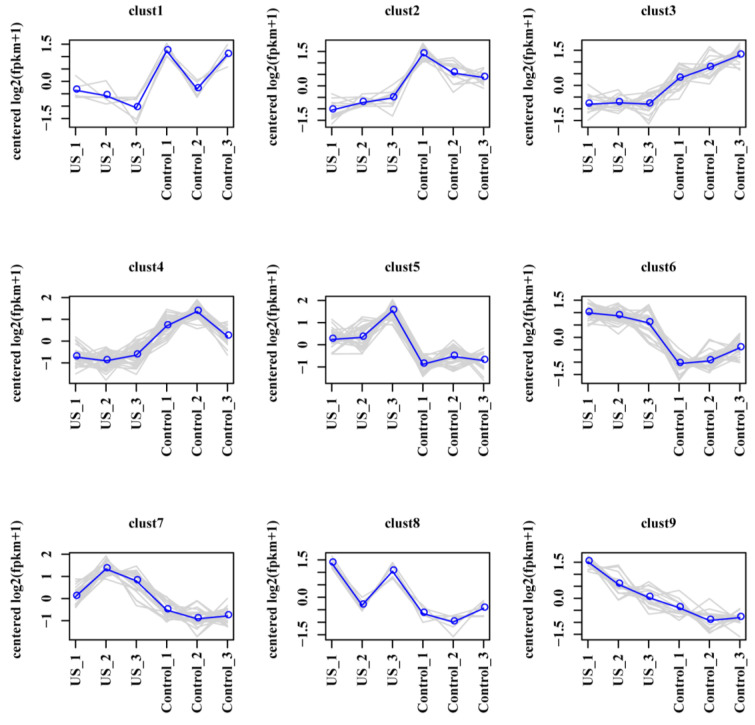
Nine expression profile classifications of DEGs in blueberry fruit. Expression profile clustering of DEGs between the control and US washing groups. The gray line shows the gene expression pattern in each cluster, while the blue line shows the average expression of all the genes in the cluster.

To further reveal the molecular mechanism of US-enhanced quality in blueberries, the RNA-Seq analyses of the control and US groups were performed. The values of Q20 (%) and Q30 (%) were above 97.87% and 93.97% after filtering, respectively (Supplementary Table S2). The clustering heat map displays the transcription levels of DEGs between two group samples, and US washing affected the abundance of DEGs (Figure 3A). The average gene expression distribution in the control and US treatment samples are presented by the violin plot, and the overall transcription abundance is well (Figure 3B). The volcano graph exhibits that a total of 163 DEGs (99 up-regulated, 64 down-regulated) were screened between the control and US treatment groups (Figure 3C and Figure 4). Meanwhile, the clustering results showed that the nine expression profile clusters were identified from DEGs based on different expression patterns (Figure 4). The numbers of DEGs from clust1 to clust1 were 5, 11, 18, 30, 28, 26, 24, 6, and 15, respectively.

### 3.4. Go Functional Analysis and KEGG Pathway Analysis of Blueberry Fruit

**Figure 5:**
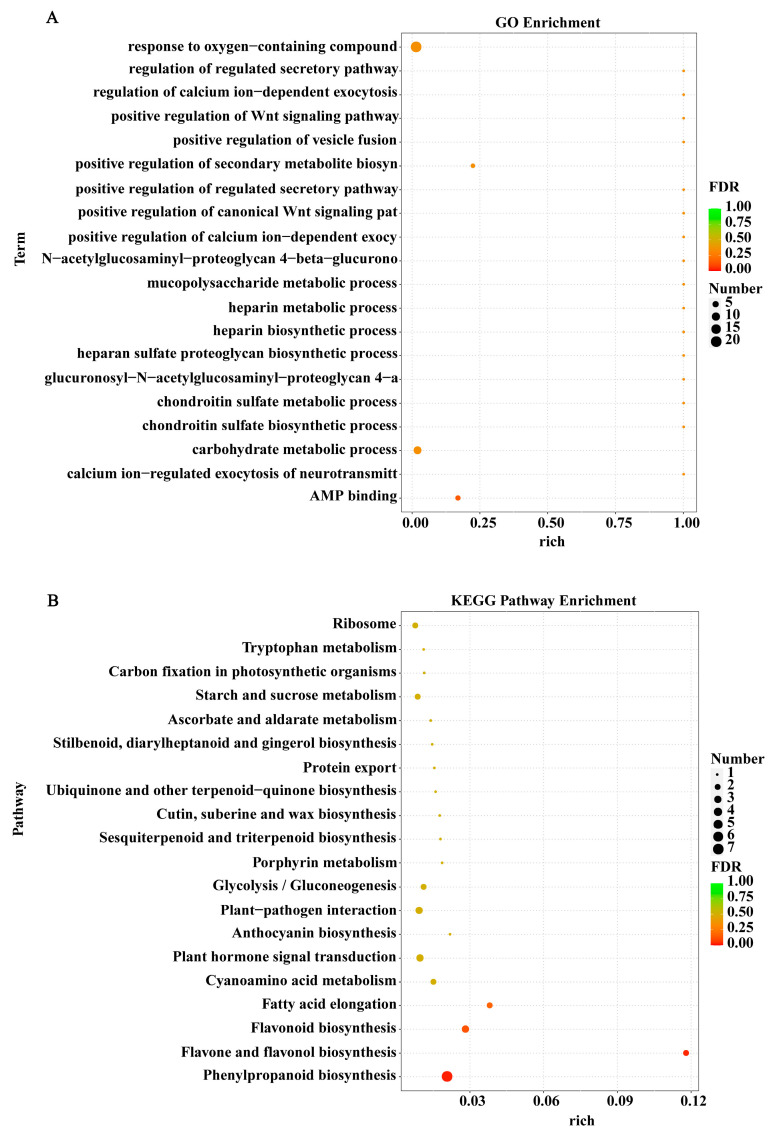
GO (**A**) and KEGG (**B**) pathway analyses of DEGs in blueberry fruit. The top 20 pathways with the most significant enrichment of GO and KEGG analyses were shown.

As shown in Figure 5, GO and KEGG analyses of 163 DEGs in blueberries were further conducted. The results displayed that the GO terms were primarily enriched in the three major categories of the response to the oxygen-containing compound, carbohydrate metabolic process, and AMP binding, respectively (Figure 5A). The KEGG analysis showed that the DEGs were primarily enriched in phenylpropanoid biosynthesis, flavonoid biosynthesis, plant–pathogen interaction and hormone signal transduction pathways (Figure 5B). These results revealed that the enhanced quality of US-treated fruit was closely related to the regulatory role of US in these biological function and metabolic pathways.

### 3.5. Candidate DEGs Involved in Transcription Factors (TFs) and Lignin Biosynthesis

**Figure 6:**
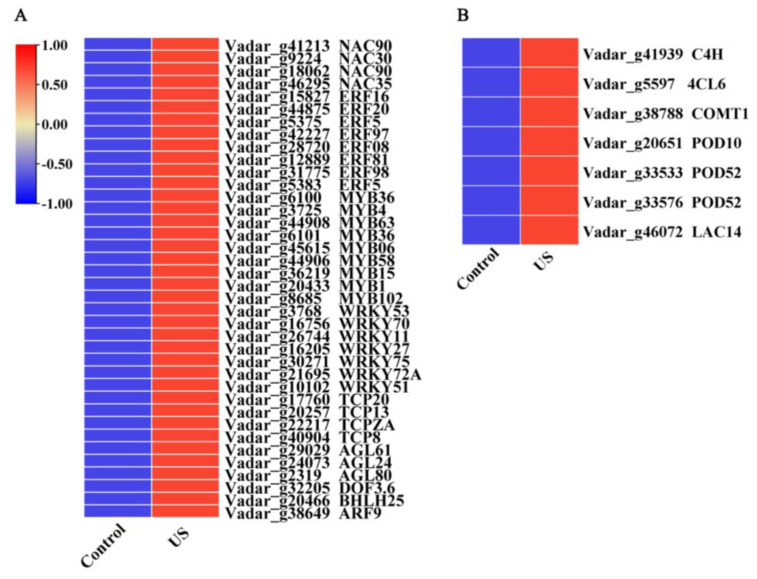
Heat maps exhibiting the expression profiles of DEGs related to TFs and lignin biosynthesis pathway. (**A**) Heat map of differentially expressed TFs. (**B**) Heat map of DEGs related to phenylpropanoid metabolism. US represents the ultrasound washing group.

The relationship between TFs and fruit quality is a scientific problem worthy of attention in blueberry fruit. Thus, to further explore the mechanism of improving fruit quality via US washing, TFs were specifically investigated, which are potentially involved in the modulation of fruit quality. As illustrated in Figure 6A, a total of nine classes of up-regulated TFs via US were screened from the DEGs, including 4 *NACs*, 8 *ERFs*, 9 *MYBs*, 7 *WRKYs*, 4 *TPCs*, 3 *AGLs*, 1 *DOF*, 1 *bHLH*, and 1 *ARF*, indicating that these transcription factors might participate in the US-mediated downstream regulation of blueberries during quality deterioration.

In addition, a heat map was also constructed based on KEGG analysis to display the expression patterns of DEGs related to lignin biosynthesis pathways. Some DEGs involved in lignin biosynthesis were markedly induced by US washing in blueberry fruit, including *C4H*, *4CL6*, *COMT1*, *POD10*, *POD52*, and *LAC14* (Figure 6A), implying that lignin biosynthesis in blueberry fruit was promoted by US washing after harvest.

### 3.6. The Effects of US Washing on the Expression of C4H, COMT1, and POD52 in Blueberries

**Figure 7:**
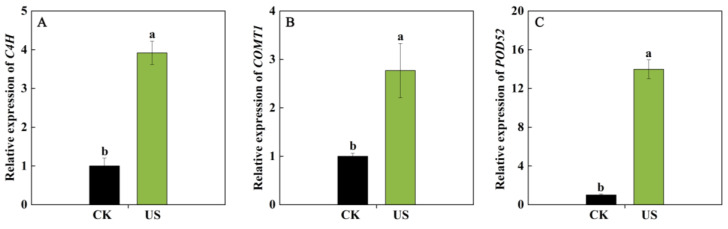
The expression of *C4H* (**A**), *COMT1* (**B**), and *POD52* (**C**) of blueberries. CK and US represent the control group and ultrasound washing group, respectively. Vertical bars represent the SD of the mean (*n* = 3). The significant differences (*p* < 0.05) between the two groups are denoted by different letters.

Among the lignin biosynthesis-related DEGs mentioned above, *C4H* (Vadar_g41939), *COMT1* (Vadar_g38788), and *POD52* (Vadar_g33533) were up-regulated by US more significantly and showed high FPKM values in blueberries. Hence, these genes were chosen to measure their transcription level through qRT-PCR. As displayed in Figure 7, US washing remarkably up-regulated the transcription levels of *C4H*, *COMT1*, and *POD52*, which were 2.87, 1.76, and 13.93 folds higher of US-treated fruit than those in control, respectively, indicating that US enhanced lignin content through activating the expression of *C4H*, *COMT1* and *POD52* genes.

### 3.7. Correlation Analysis

**Figure 8:**
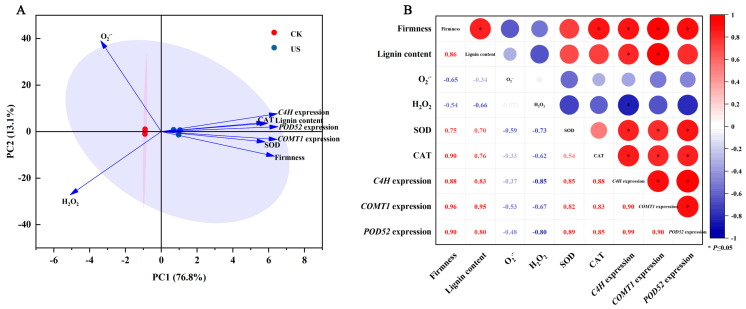
The correlation analysis of US-improved quality in postharvest blueberry fruit. (**A**) PCA of US treatment impacts the quality of blueberry fruit. (**B**) Correlation analysis of US washing impacts on the quality of blueberry fruit. CK and US represent the control group and ultrasound washing group, respectively. Red color and blue color indicated a positive correlation and a negative correlation, respectively. The asterisk * represents a significant correlation (*p* < 0.05).

As shown in Figure 8A, a PCA of relevant indicators in blueberry fruit was performed. The contribution rate of PC1 and PC2 were 13.1% and 76.8%, respectively. Additionally, significant separations could be observed between the US-treated fruit and control fruit.

The correlation analysis of firmness, lignin content, O_2_^·−^ production rate, H_2_O_2_ content, SOD activity, CAT activity, *C4H*, *COMT1*, and *POD52* expression of blueberry fruit was conducted as shown in Figure 8B. The O_2_^·−^ production rate and H_2_O_2_ content were negatively correlated with the activities of SOD and CAT activity in blueberry fruit. Additionally, the firmness showed significantly positive correlations (*p* < 0.05) with lignin content, which positively associated (*p* < 0.05) with the expression of *C4H*, *COMT1*, and *POD52* genes. These results indicated that the positive role of US treatment in maintaining the firmness of blueberry fruit was closely related to its activation of lignin biosynthesis genes to induce lignin deposition.

## 4. Discussion

US, as a non-thermal technology, could maintain firmness, increase antioxidant activity, and inhibit microbiological decay in postharvest horticultural crops, such as strawberry fruit [12] and freshly cut cauliflower [8]. It has been revealed that firmness acts as a crucial indicator for assessing fruit softening, which directly impacts fruit quality and consumer acceptance [26]. For blueberry fruit, previous studies have confirmed that US washing could not only reduce the microorganism’s growth but also maintain fruit quality via enhancing firmness and antioxidant capacity [13,14]. Similarly, our study found that US washing could maintain higher firmness and lignin content in blueberries (Figure 1), contributing to the quality maintenance in blueberry fruit. However, the underlying mechanism of the ultrasound-enhanced fruit quality of blueberries is still unknown.

Recent studies confirmed that fruit softening and decay primarily resulted from the membrane lipid peroxidation caused by overproduction of ROS, such as O_2_^·−^ and H_2_O_2_ [27]. The efficient functioning of antioxidant enzymes, like SOD, CAT, and ascorbate peroxidase (APX), can scavenge ROS accumulation, thus alleviating oxidative damage in postharvest fruits [28]. In this study, US washing could significantly enhance SOD and CAT activity in blueberry fruit compared with the control, accompanied by a reduced O_2_^·−^ production rate and H_2_O_2_ content (Figure 2). It could be concluded that the function of low-frequency US in stimulating the antioxidant activity was benefit to protecting the fruit against oxidative damage [14]. Similar results were also reported in jujubes [29] and cherry tomatoes [30] treated with US. Chen et al. [31] also revealed that retaining higher activities of SOD, CAT, and APX contributed to reducing ROS accumulation and alleviating oxidative damage, thereby extending the shelf life of postharvest blueberries. These findings suggested that the protection of cell membranes via US washing might be attributed to the enhancement of antioxidant enzymes to retain ROS balance in blueberry fruit.

Texture is an important quality characteristic of postharvest fruits, among which softening is one of the most striking and irreversible features, impacting quality and consumer acceptability [32]. Furthermore, the lignin deposition causing the increase in fruit firmness is another important phenomenon of textural change in postharvest fruits. Lignin is recognized to synthesize through the branch of the phenylpropanoid pathway. A series of genes (such as *PAL*, *C4H*, *4CL*, *COMT*, *CAD*, *POD*, and *LAC*) encoding lignin synthesis-related enzymes are involved in this process, and these genes were regulated via upstream transcriptional regulators [32,33,34]. In loquat fruit, it has been confirmed that AP2/ERFs, MYBs, NACs, bHLHs, MADS, and CAMTA3 could regulate the expression of *EjPAL1*, *Ej4CL1*, *EjCADs*, *EjPODs*, and *EjLAC12* to impact the lignin biosynthesis during cold storage [35,36,37,38]. In pear fruit, PbrMYB24 participated in lignin biosynthesis via modulating the transcription of *4CL1*, *CCOAMT1*, and *CESA8b* [31]. CgMYB58 could directly bind to the promoters of *CgPAL1*, *CgPAL2*, *Cg4CL1*, and *CgC3H* genes, thereby activating their expressions to promote lignin biosynthesis in postharvest pummelo fruit [34]. These studies demonstrated that the textural change in fruits caused by lignin biosynthesis is a complicated process, and several TFs and structural genes took part in the modulation process. In this work, RNA-Seq analysis showed that nine TF families genes containing 4 *NACs*, 8 *ERFs*, 9 *MYBs*, 7 *WRKYs*, 4 *TPCs*, 3 *AGLs*, 1 *DOF*, 1 *bHLH*, and 1 *ARF*, were induced via US washing treatment in blueberries (Figure 6A). Among these differentially expressed TFs, NAC, ERF, MYB, and bHLH have been widely confirmed to be involved in the lignin biosynthesis of postharvest fruits to influence fruit texture [32], indicating that they might be exerting an important role in regulating the lignin biosynthesis process in blueberries. Moreover, the gene expression of *C4H*, *4CL6*, *COMT1*, *POD10*, *POD52*, and E*jLAC12* in blueberry fruit were significantly up-regulated via US washing (Figure 6B), and these increases coincided with the higher lignin content and firmness in US-treated fruit (Figure 1), implying that the induced lignin biosynthesis-related genes promoted lignin accumulation. The expression levels of *C4H*, *COMT1*, and *POD52* were also carried out via qRT-PCR, and US washing could remarkably up-regulate *C4H*, *COMT1*, and *POD52* expressions, which were in agreement with the results of RNA-Seq data. Combining with the results of the positive correlation between firmness, lignin content, and the expression of *C4H*, *COMT1*, and *POD52* genes, it could be speculated that these three genes might play a key role in modulating the lignin biosynthesis of blueberry fruit. Similarly, Qu et al. [39] revealed that the accumulation of phenolic and lignin was primarily due to the up-regulating expression of phenylpropane metabolism-related genes, such as *VaPAL*, *VaC4H*, *Va4CL*, *VaCAD*, *VaPPO*, and *VaPOD*, in blueberry fruit during storage. Yang et al. [40] also reported that VcMYB4a could down-regulate the transcription levels of *Vc4CL5/7*, *VcCOMT1/2*, and *VcCAD1/2* genes, thus participating in the lignin biosynthesis of blueberry fruit. Taken together, it could be inferred that US-induced higher firmness of blueberries was attributed to the lignin deposition, which might be associated with the activated expression of TFs (*NACs*, *ERFs*, *MYBs*, and *bHLH*) and downstream genes (*C4H*, *COMT1*, and *POD52*). In the future, we can continue to explore the molecular mechanism of an US, promoting lignin synthesis at the transcriptional regulation level.

## 5. Conclusions

In summary, US washing could improve the fruit quality of postharvest blueberries, as manifested in increased firmness and lignin content. Meanwhile, US washing reduced the accumulation of ROS via promoting SOD and CAT activities, which was beneficial for the maintenance of quality in blueberry fruit. Moreover, RNA-Seq analysis identified the potential TFs and structural genes related to lignin biosynthesis, which played a crucial role in the enhancement of lignin content via US washing, thereby maintaining higher firmness in blueberry fruit. Our findings lead to a better understanding of mechanisms underlying US-improved fruit quality at the molecular level.

## Data Availability

The original contributions presented in the study are included in the article/Supplementary Materials, further inquiries can be directed to the corresponding author.

## Supplementary Materials

The following supporting information can be downloaded at: https://www.mdpi.com/article/10.3390/foods13203246/s1, Table S1: Details of primers for qRT-PCR used in this study, Table S2: Summary of RNA-Seq data and sequence assembly.
