# Supplementary material for: Ultrasound as a Physical Elicitor to Improve Texture in Blueberry Fruit: Physiological Indicator and Transcriptomic Analysis
Source: Foods. 2024 Oct 12;13(20):3246. doi: 10.3390/foods13203246 (PMC11508173; doi:10.3390/foods13203246)
Supplement: Supplementary file 1 [file foods-13-03246-s001.zip › foods-3217097-Supplementary data.pdf]

Table S1. Details of primers for qRT-PCR used in this study.

Gene name	Forward primer(5'-3')	Reverse primer(5'-3')
EF1A	TGGAAATGGGTATGCCCCAG	ACCATACCGGCATCTCCATTC
C4H	TCAAGGAGAGGAGGTTGCAG	TCAAGGAGAGGAGGTTGCAG
COMT1	AATGATCCCAGATGGCACGA	TATGAACAGCACCGTGTCCT
POD52	TATGAACAGCACCGTGTCCT	TATGAACAGCACCGTGTCCT

Table S2. Summary of RNA-Seq data and sequence assembly.

sample	total clean_reads	total_map	unique_map	Q20(%)	Q30(%)
Control_1	43935034	36548513 (85.20%)	35794194 (95.08%)	97.87	93.97
Control_2	33257964	40351294 (85.76%)	27013723 (95.25%)	97.96	94.27
Control_3	44198288	36707995 (85.36%)	35637320 (94.58%)	97.97	94.29
US_1	42899472	37645647 (85.68%)	34596660 (94.66%)	97.95	94.23
US_2	47052442	28360282 (85.27%)	38284761 (94.88%)	97.91	94.06
US_3	43002488	37678660 (85.25%)	34815627 (94.84%)	97.89	94.12
